# THE IMPACT OF CHILDHOOD READING ENVIRONMENTS ON COGNITIVE HEALTH IN LATER LIFE AMONG OLDER EUROPEANS

**DOI:** 10.1093/geroni/igae098.0402

**Published:** 2024-12-31

**Authors:** Haosen Sun, Yueming Xi

**Affiliations:** University of Nevada, Reno, Reno, Nevada, United States; McMaster Univeristy, Hamilton, Ontario, Canada

## Abstract

While educational achievement helps to accumulate cognitive reserve and promote cognitive function in later life, the influence of the early living environment before completing formal education remains unclear. This study aims to investigate how childhood reading environments, alongside educational achievements, jointly contribute to cognitive health in older adulthood. Analyzing life history data from 123,199 older Europeans aged 50 and above, sourced from the Survey of Health, Ageing and Retirement in Europe, this research examines the interplay between childhood reading environments and educational achievement and their joint effect on cognitive health at older ages. Having more books in the household at age 10 is positively associated with higher cognitive functioning in later life, independent of educational achievements and other childhood socioeconomic status (SES) factors like household size and number of rooms. Additionally, access to books in childhood partially alleviates the cognitive disparities associated with educational achievement. These findings indicate that a rich reading environment at home in childhood offers enduring benefits for cognitive health, which is particularly valuable for individuals who ended up with lower educational achievements. This underscores the potential of interventions focused on enhancing early reading environments to promote long-term cognitive resilience.

